# Crystal structures of butyl 2-amino-5-hy­droxy-4-(4-nitro­phen­yl)benzo­furan-3-carboxyl­ate and 2-meth­oxy­ethyl 2-amino-5-hy­droxy-4-(4-nitro­phen­yl)benzo­furan-3-carboxyl­ate

**DOI:** 10.1107/S205698901900728X

**Published:** 2019-05-24

**Authors:** Rosita Diana, Angela Tuzi, Barbara Panunzi, Antonio Carella, Ugo Caruso

**Affiliations:** a Department of Agriculture, University of Napoli Federico II, Via Università, 100, 80055 Portici NA, Italy; b Department of Chemical Sciences, University of Napoli Federico II, Via Cintia, 80126 Napoli, Italy

**Keywords:** crystal structure, benzo­furan, anti tumoral properties

## Abstract

The title benzo­furan derivatives 2-amino-5-hy­droxy-4-(4-nitro­phen­yl)benzo­furan-3-carboxyl­ate (BF1), C_19_H_18_N_2_O_6_, and 2-meth­oxy­ethyl 2-amino-5-hy­droxy-4-(4-nitro­phen­yl)benzo­furan-3-carboxyl­ate (BF2), C_18_H_16_N_2_O_7_, recently attracted attention because of their promising anti­tumoral activity. BF1 crystallizes in the space group *P* . BF2 in the space group *P*21/*c*. The nitro­phenyl group is inclined to benzo­furan moiety with a dihedral angle between their mean planes of 69.2 (2)° in BF1 and 60.20 (6)° in BF2. A common feature in the mol­ecular structures of BF1 and BF2 is the intra­molecular N—H⋯O_carbon­yl_ hydrogen bond. In the crystal of BF1, the mol­ecules are linked head-to-tail into a one-dimensional hydrogen-bonding pattern along the *a*-axis direction. In BF2, pairs of head-to-tail hydrogen-bonded chains of mol­ecules along the *b*-axis direction are linked by O—H⋯O_meth­oxy_ hydrogen bonds. In BF1, the butyl group is disordered over two orientations with occupancies of 0.557 (13) and 0.443 (13).

## Abstract 2

There are small differences in the mol­ecular geometries of the title compounds regarding the orientation of meth­oxy­ethyl or butyl group. Common features in the two mol­ecular structures are the presence of an intra­molecular N—H⋯O_carbon­yl_ hydrogen bond and the inclination of nitro­benzene ring to the benzo­furan mean plane.

## Chemical context   

Organic heterocyclic materials play a very important role in the field of synthetic chemistry because of their relevant biological activity: the great majority of marketed drugs contain at least one heterocycle in their mol­ecular structure (Wu, 2012 ▸; Gomtsyan, 2012 ▸). At the same time, the high polarizability of heterocycles results in particular optical and electronic properties that make these systems key elements in materials chemistry, fundamental for the rapid development of new advanced materials. Heterocyclic-based novel materials have been investigated in the fields of organic photovoltaics (Maglione *et al.*, 2017 ▸; Maglione, Carella, Centore *et al.*, 2016 ▸; Maglione, Carella, Carbonara *et al.*, 2016 ▸; Holliday *et al.*, 2016 ▸; Jin & Irfan, 2017 ▸; Bruno *et al.*, 2014 ▸; Morvillo *et al.*, 2016 ▸), luminescent materials (Caruso *et al.*, 2013 ▸; Borbone *et al.*, 2016 ▸) non-linear optics (Carella *et al.*, 2005 ▸; Caruso *et al.*, 2006 ▸). Among compounds containing oxygen heterocycles, benzo­furan derivatives have proven to be powerful systems displaying a wide range of biological properties including anti­microbial (Alper-Hayta *et al.*, 2008 ▸; Piotto *et al.*, 2017 ▸; Soni & Soman, 2014 ▸), anti­tumor (Xie *et al.*, 2015 ▸; Hayakawa *et al.*, 2004 ▸), anti-parasitic (Thévenin *et al.*, 2013 ▸)and analgesic activities (Wang *et al.*, 2017 ▸). In the field of materials chemistry, benzo­furan derivatives have found applications in the area of industrial dyes as optical whiteners or disperse dyes characterized by high fastness properties. Moreover, inter­esting applications of benzo­furan-based organic sensitizers for dye-sensitized solar cells (Justin Thomas & Baheti, 2013 ▸) have been recently discovered. Different synthetic strategies are reported for the synthesis of benzo­furans, the majority of which are associated with transition-metal-catalysed annulation reactions of pre-functionalized substrates that are typically synthesized by Heck or Sonogashira coupling reactions (Anderson *et al.*, 2006 ▸; Guo *et al.*, 2009 ▸; Li *et al.*, 2011 ▸; Yue *et al.*, 2005 ▸). In a recent paper, inspired by a previous work (Obushak, 2002 ▸), we managed to fine-tune a synthetic procedure for the synthesis of two benzo­furan derivatives and their anti­proliferative activity and ability to bind telomeric DNA was proved (Carella *et al.*, 2019 ▸). This synthesis was realized by using a cheap and simple reaction known as the Craven reaction, which does not need either a precious transition metal as catalyst or an inert gas environment to be carried on. The Craven reaction is a well-known procedure for the synthesis of benzodi­furan derivatives that consists of the reaction of 1,4-benzo­quinone with various cyano­acetic esters in alcoholic ammonia (King & Newall, 1965 ▸; Caruso *et al.*, 2009 ▸; Carella *et al.*, 2012 ▸). While the Craven reaction typically affords benzodi­furan derivatives almost exclusively, we observed (Carella *et al.*, 2019 ▸) that, by properly optimizing the reaction conditions, it is possible to isolate benzo­furan deriv­atives as the main product and in significant yields (up to 38%). The formation of benzo­furan derivatives was confirmed by elemental CHN analysis.
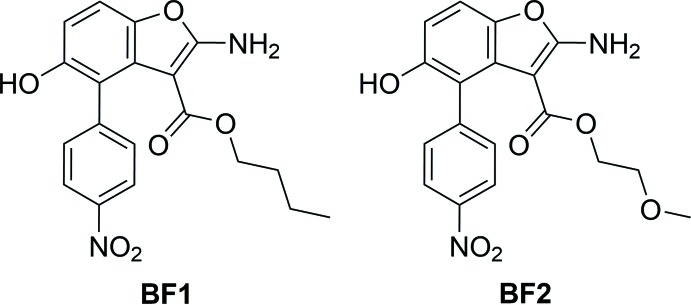


**Figure 1:**
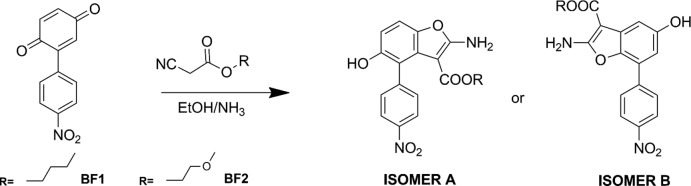
Reaction scheme for the preparation of benzo­furan derivatives BF1 and BF2: the reaction could afford two different isomers.

As shown in Fig. 1 ▸, two different constitutional isomers can in principle form during the reaction, with the *p-*nitro­phenyl group functionalizing the benzo­furan ring in position 4 (isomer *A*) or 7 (isomer *B*). The NMR analysis and the differential scanning calorimetry (DSC) analysis performed suggested that only one of the two possible isomers was recovered for both of the benzo­furan derivatives. In particular, following the results of NMR analysis, in the previous paper we proposed that type *A* isomers were obtained, namely the title compounds. The determination of the real mol­ecular structure of the isomer actually formed during the reaction is undoubtedly also inter­esting in consideration of the anti-tumoral properties shown by this class of compounds (Carella *et al.*, 2019 ▸). In this context, to ultimately confirm that the *A* isomer forms, we report here the structural investigation of the previously synthesized benzo­furan derivatives BF1 and BF2.

## Structural commentary   

**Figure 2:**
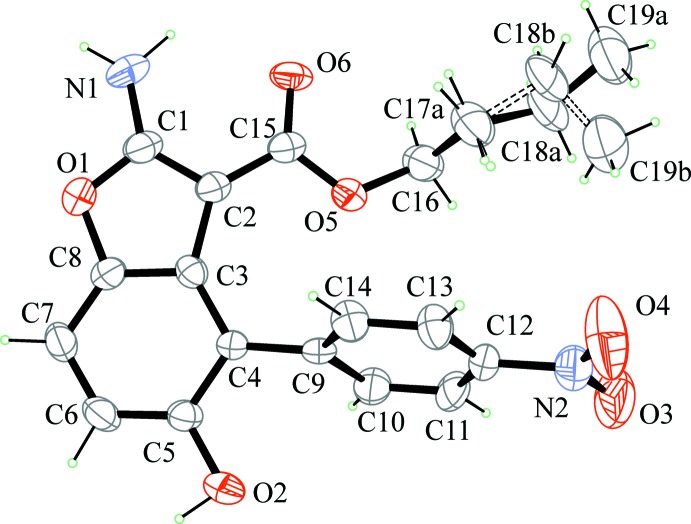
Mol­ecular structure of BF1, with displacement ellipsoids drawn at the 30% probability level. The minor component of the disordered butyl group is drawn with open bonds.

XRD analysis of single crystals grown as described in the experimental section confirmed that the benzo­furan derivatives previously reported (Carella *et al.*, 2019 ▸) are the isomers *A* indicated in Fig. 1 ▸. The mol­ecular structures of BF1 and BF2 are shown in Figs. 2 ▸ and 3 ▸. The obtained isomers are characterized by a *cisoid* configuration of the two substituents at C2 and C4, with a higher steric hindrance as compared to isomer *B*: in this case, the *ortho*-orientating effect of the electron-acceptor nitro­phenyl group drives the path of the reaction, prevailing over steric considerations.

**Figure 4:**
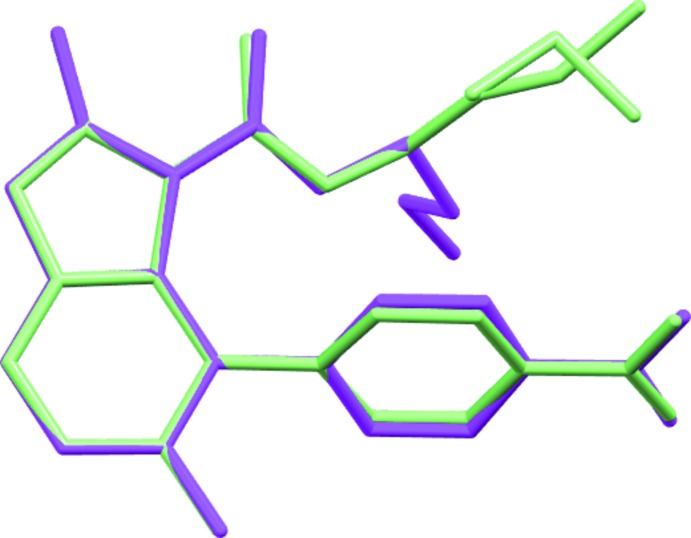
Superimposition of BF1 (light green) with BF2 (magenta) mol­ecules showing the different orientation of the ester groups. H atoms are not included for clarity. The two positions of the disordered butyl group are shown for BF1.

No unusual geometric features were found in either structure, all bond lengths and angles being in expected ranges and in agreement with analogous benzo­furan derivatives reported in the *Database survey* section of this paper. A common structural feature in BF1 and BF2 is the intra­molecular N—H⋯O hydrogen bond between the amine group and the carbonyl oxygen (Tables 1 ▸ and 2 ▸) that leads to near co-planarity of the –COO– group and the benzo­furan ring [15.71 (18)° in BF1 and 23.85 (2)° in BF2]. The geometry at N1 amine atom is almost planar [deviation of N1 from least square plane of attached atoms is 0.01 (4) Å in BF1 and 0.16 (2) Å in BF2]. A shortening of the N1—C1 bond distance [1.318 (7) Å in BF1 and 1.335 (3) Å in BF2] is observed, compared with a mean value for a C*sp* ^2^—NH_2_ bond of 1.336 (17) Å (Allen *et al.*, 1987 ▸). Such geometric features suggest a partial conjugation of the N atom with benzo­furan that is more marked in BF1, where a shorter N1—C1 bond distance and a more evident planar geometry at N1 are found. The benzo­furan group is planar within 0.049 (4) Å in BF1 and 0.040 (2) Å in BF2; the nitro­benzene group is planar within 0.027 (5) Å in BF1 and 0.074 (2) Å in BF2. The dihedral angle between benzo­furan and nitro­phenyl mean planes is 69.26 (16)° in BF1 and 60.20 (6)° in BF2. The orientation of the nitro­phenyl group clearly minimizes inter­actions with the adjacent ester group. Small differences (Fig. 4 ▸) are found between the mol­ecular geometries of BF1 and BF2, apart from the different orientation of the meth­oxy­ethyl or butyl groups resulting from a different torsion angle around C16—C17 [mean value of 172.9 (11)° in BF1 and 83.6 (2)° in BF2]. In BF1, the butyl group is disordered over two orientations that differ in the torsion angle around C17—C18 bond [C16—C17*A*—C18*A*—C19*A* = 171.5 (17)° and C16—C17*B*—C18*B*—C19*B* = −81 (2)°].

## Supra­molecular features   

In BF1 and BF2, the crystal packing is dominated by strong N—H⋯O and O—H⋯O hydrogen bonds and weak C—H⋯O inter­actions (Tables 1 ▸ and 2 ▸). Weak intermolecular C—H⋯π interactions are also present in BF2 due to the edge-to-face contacts between nitrobenzene and furan ring systems.

**Figure 5:**
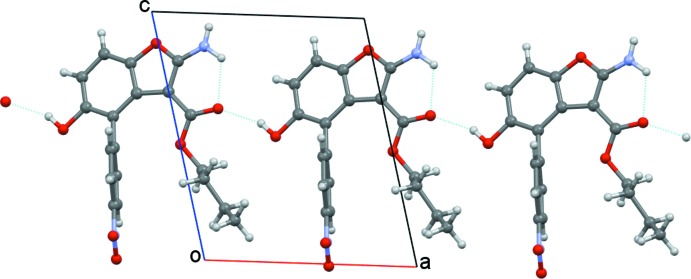
Partial crystal packing of BF1 showing a chain of head-to-tail hydrogen-bonded mol­ecules (intra- and inter­molecular hydrogen bonds are indicated by cyan dashed lines). Only the major component of the disordered buthyl group is shown.

In BF1, the amine group is involved only in one intra­molecular hydrogen bond, acting as donor towards the close carbonyl O atom. The hy­droxy group is involved only in one inter­molecular hydrogen bond, acting as donor towards the carbonyl O atom of an adjacent mol­ecule. In the crystal packing, chains of strong O—H⋯O head-to-tail hydrogen-bonded mol­ecules are formed along *a*-axis direction (Fig. 5 ▸). The chains are connected into a three-dimensional network by weak inter­molecular inter­actions involving the nitro group as acceptor from C_ar_—H atoms (Table 1 ▸). In particular, the C11 atom acts as a hydrogen-bond donor to the nitro O3 atom, forming centrosymmetric dimers.

**Figure 6:**
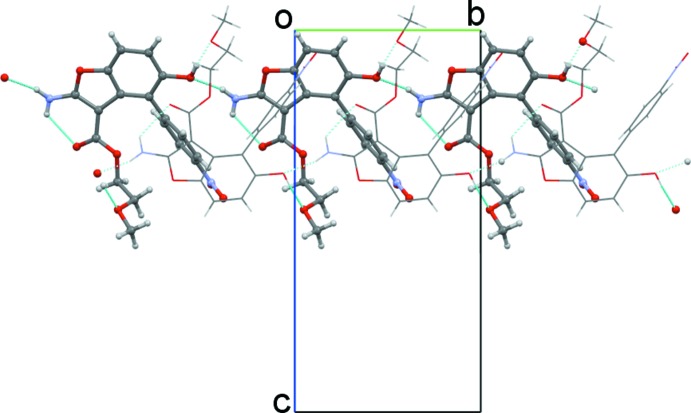
Partial crystal packing of BF2 showing two head-to-tail hydrogen-bonded chains connected by O—H⋯O_meth­oxy_ hydrogen bonds (the deeper chain is drawn in capped stick style; intra- and inter­molecular hydrogen bonds are indicated by cyan dashed lines).

In BF2, one more O acceptor atom is present compared to BF1. The amine group is involved both in intra- and inter­molecular hydrogen bonds. Similarly to BF1, an intra­molecular N—H⋯O hydrogen bond is formed with the carbonyl oxygen atom. An inter­molecular N—H⋯O hydrogen bond is formed with the hy­droxy oxygen atom of an adjacent mol­ecule as acceptor. The hy­droxy group is also involved as donor in O—H⋯O hydrogen bonds with the meth­oxy O atom of an adjacent mol­ecule. In the crystal packing, neighbouring head-to-tail hydrogen-bonded chains of mol­ecules are linked through O—H⋯O_meth­oxy_ hydrogen bonds and weak intermolecular C—H⋯π(benzofuran) interactions, wrapping around the 2_1_ screw axis (Fig. 6 ▸).

## Database survey   

A search of the Cambridge Structural Database (CSD, Version 5.40, November 2018 with February 2019 updates; Groom *et al.*, 2016 ▸) found 25 structures that match the fragment made of benzo­furan substituted at the 2-position with –N*X*_2_ (*X* = C, H). The hits found are crystal structures determined at temperatures in the range 103–298 K. Among these, 11 structures match the 2-amino-benzo­furane fragment present in BF1 and BF2: DOZYIB (Caruso *et al.*, 2009 ▸), FERXEG (Otsuka *et al.*, 2004 ▸), FUFBEO (Murai *et al.*, 2004 ▸), GOWHEF (Tandel *et al.*, 1998 ▸), GUYXEE (Yi *et al.*, 2010 ▸), QINXUI (Roviello *et al.*, 2013 ▸), RAMZAH and RAMZEL (Ishikawa *et al.*, 2005 ▸), RISSAP and RISSET (Li *et al.*, 2014 ▸) and SECDUZ (Becker *et al.*, 1989 ▸). Of these, two are similar to the title compounds: 2-amino-3-(p-tol­yl)benzo­furan-4-yl acetate and 2-amino-3-(4-meth­oxy­phen­yl)benzo­furan-4-yl acetate (RAMZEL and RAMZAH) in which the aryl ring is inclined to the benzo­furan ring system by 61.9 (5)° and 52.1 (6)°, respectively [69.26 (16)° in BF1 and 60.20 (6)° in BF2]. The acetate group is inclined to the benzo­furan ring system by 68.8 (6)° in RAMZAH and 75.68°(5) in RAMZEL, while in the title compounds near co-planarity of the –COO– group with benzo­furan is observed [15.71 (18)° in BF1 and 23.85 (2)° in BF2]. In the 11 hits, the C—N_amine_ bond distance ranges between 1.305 and 1.408 Å with an average value of 1.34 (2) Å, compared to 1.318 (7) Å in BF1 and 1.335 (3) Å in BF2.

## Analysis of Hirshfeld surfaces and inter­action energies   

In order to detect additional packing features and to analyse close inter­molecular contacts in BF1 and BF2, we have examined the Hirshfeld surfaces and two-dimensional fingerprint plots using *CrystalExplorer17.5* (Turner *et al.* 2017 ▸). The electrostatic potentials were calculated using *TONTO*, integrated within *CrystalExplorer*. The inter­action energies between the mol­ecules were obtained using wavefunctions at the B3LYP/6-31G(d,p) level. The total inter­action energy was calculated for a 3.8 Å radius cluster of mol­ecules around the selected mol­ecule. The scale factors used in the CE-B3LYP benchmarked energy model (Mackenzie *et al.* 2017 ▸) are given in footnote of Tables 3 ▸ and 4 ▸. Calculations were made for both disorder components of BF1; results for the major disordered component (named BF1-molA) are reported since very small differences were found between them.

**Figure 7:**
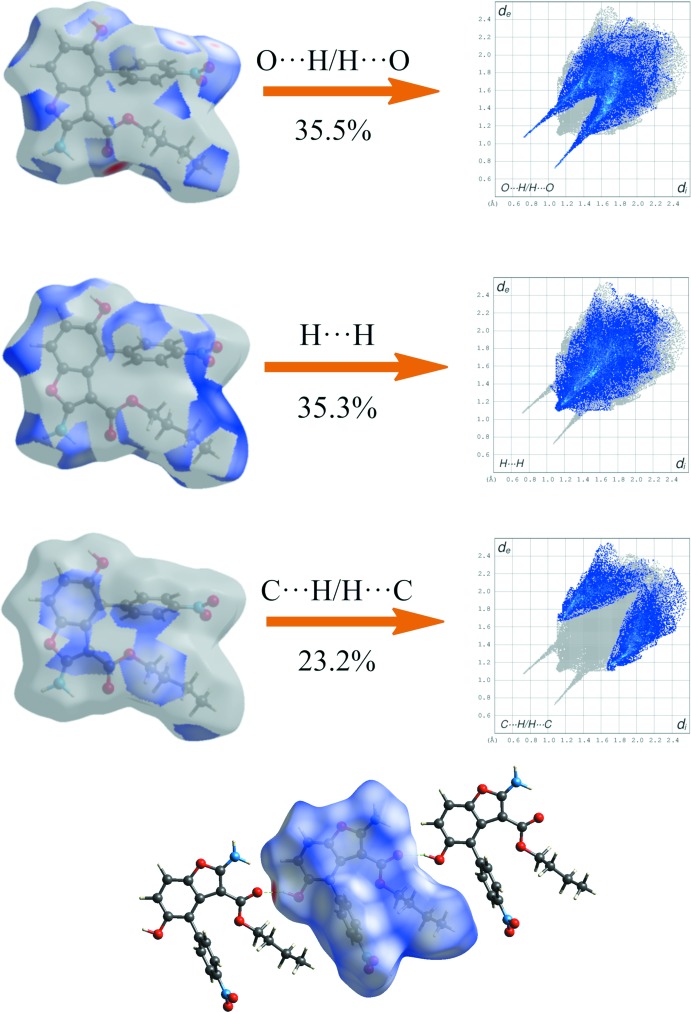
Two-dimensional fingerprint plots of significant inter­molecular contacts for BF1 (major disorder component) with the *d*_norm_ surfaces indicating the relevant surface patch associated with the specific contact. The Hirshfeld surface mapped over *d*_norm_ with strong hydrogen bonded mol­ecules outside is also shown.

**Figure 8:**
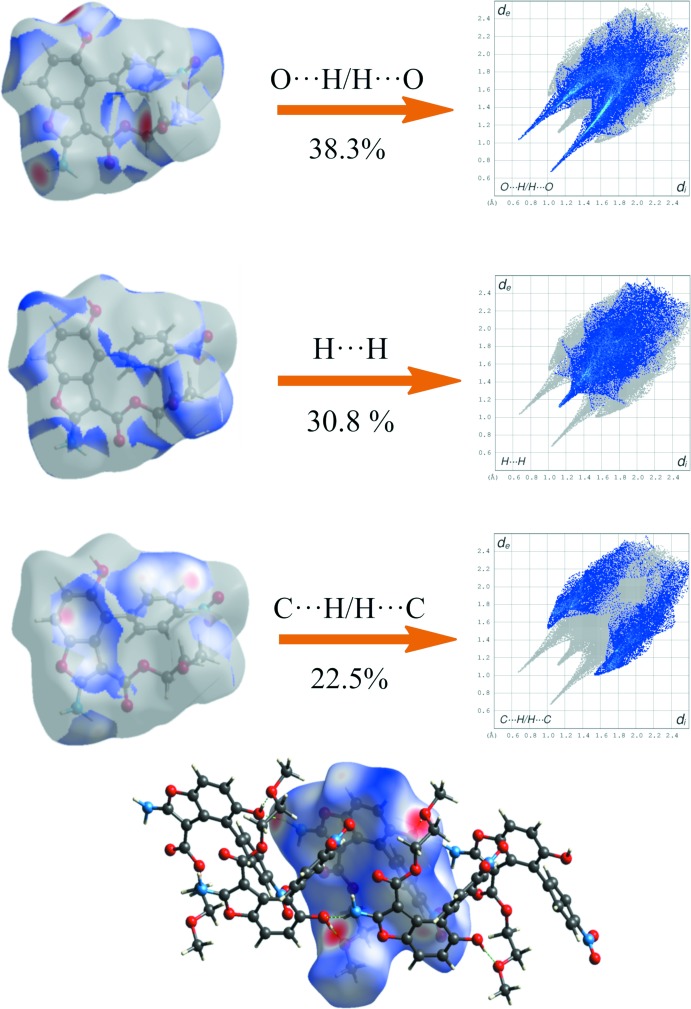
Two-dimensional fingerprint plots of significant inter­molecular contacts for BF2 with the *d*_norm_ surfaces indicating the relevant surface patch associated with the specific contact. The Hirshfeld surface mapped over *d*_norm_ with strongly hydrogen-bonded mol­ecules outside is shown.

**Figure 9:**
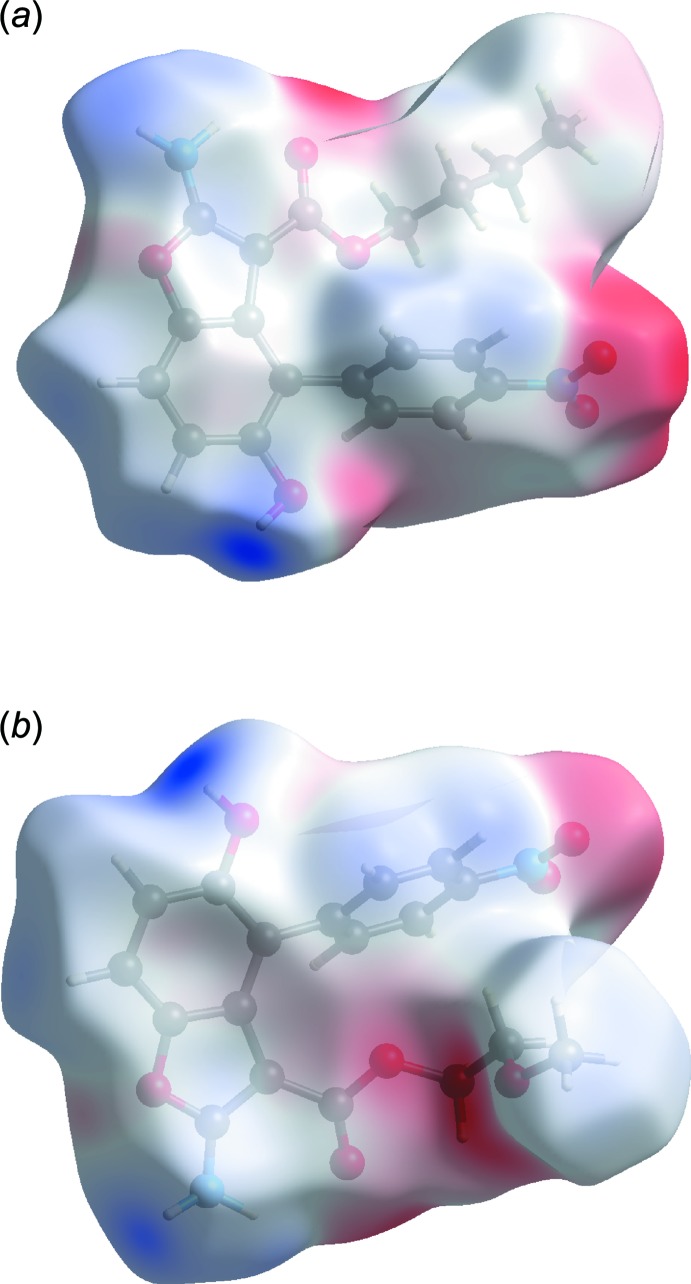
Hirshfeld surface mapped over the electrostatic potential energy for (*a*) the major disorder component of BF1 and (*b*) BF2.

The two-dimensional fingerprint plots of BF1-molA (Fig. 7 ▸) and BF2 (Fig. 8 ▸) show the significant inter­molecular inter­actions. In both compounds, the greatest contribution arises from O⋯H/H⋯O inter­actions (35.5% in BF1-molA and 38.3% in BF2) that correspond to strong hydrogen bonds (see Tables 1 ▸ and 2 ▸). These inter­actions are displayed as a pair of sharp spikes at about *d*_i_ + *d*_e_ = 2.0 Å, symmetrically disposed with respect to the diagonal in Fig. 7 ▸ *a*nd 8 (top). The large number of H⋯H inter­actions (35.3% in BF1-molA and 30.8% in BF2) are shown as a diagonal blue strip that ends, with a more evident sting in BF2, at about *d*_i_ = *d*_e_ = 1.08 Å (Figs. 7 ▸ and 8 ▸, middle). The C⋯H/H⋯C plot (23.2% in BF1-molA and 22.5% in BF2, Figs. 7 ▸ and 8 ▸, bottom) shows two broad symmetrical wings at about *d*_i_ + *d*_e_ = 3.0 Å in BF2, typical of C—H⋯π inter­actions. No significant C⋯C contacts were found in BF1 and BF2, confirming the absence of π–π stacking inter­actions. Other contacts are N⋯H/H⋯N (2.9% in BF1-molA and 2.3% in BF2); C⋯O/O⋯C (1.5% in BF1-molA and 1.8% in BF2); O⋯O (1.7% in BF1-molA and 2.6% in BF2). In the Hirshfeld surfaces of BF1 and BF2 mapped over *d*_norm_ (Figs. 7 ▸ and 8 ▸), the strong inter­molecular hydrogen bonds are observed as red spots. These inter­actions can be also identified in the Hirshfeld surfaces mapped over the electrostatic potential (Fig. 9 ▸) where the negative potential around oxygen appear as bright red and positive potential around hydrogen as bright blue.

The energies of inter­action between mol­ecules in the crystal structures of BF1-molA and BF2 were explored using *CrystalExplorer* to perform energy calculations for a 3.8 Å cluster of mol­ecules around the selected mol­ecule. The data reported in Tables 3 ▸ and 4 ▸ show that the crystal packing in both compounds is mostly stabilized by electrostatic and dispersion energy and that the major contribution to the electrostatic energy originates from strong hydrogen bonds. Some inter­action energies were analysed and their possible inter­action energies and geometry are reported. In Table 3 ▸, the lowest *E*_ele_ inter­action energies correspond to pairs of mol­ecules involved in the inter­molecular hydrogen bonds reported in Table 1 ▸ and to weak N—H⋯O—N and C—H⋯O=C inter­actions (not included in Table 1 ▸ because the donor—H⋯acceptor geometry is out of the normal range). One destabilizing positive inter­action energy (*E*_ele_ = 8.5 KJ mol^−1^) can be associated with a pair of mol­ecules where the nitro groups point to each other with repulsive N—O⋯O—N inter­actions. In Table 4 ▸, the analysed inter­actions with low *E*_ele_ inter­action energies can be associated with pairs of hydrogen-bonded mol­ecules (Table 2 ▸).

**Figure 10:**
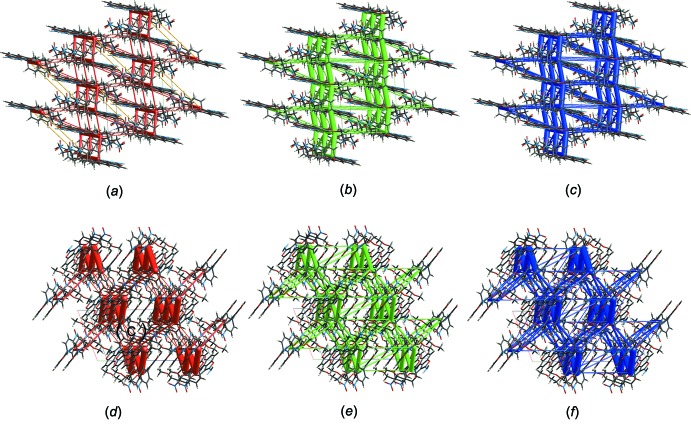
Framework inter­actions in BF1-molA (*a*, *b*, *c*) and in BF2 (*d*, *e*, *f*) with the electrostatic energy (red cylinders), dispersion energy (green cylinders) and total energy (blue cylinders). Yellow cylinders in (*a*) depict destabilizing positive inter­action energies. Scale for tube/cylinder size is 80, cutoff of 8.00 kJ mol^−1^ used.

The supra­molecular architectures for the crystal structures of BF1 and BF2 (Fig. 10 ▸) were visualized by energy framework calculations (Turner *et al.*, 2015 ▸; Mackenzie *et al.*, 2017 ▸) that were performed using CE-B3LYP energy model for a 2 x 2 x 2 (BF1-molA) and a 2 x 2 x 1 (BF2) block of unit cells. Energies between mol­ecular pairs are represented as cylinders joining the centroids of pairs of mol­ecules, with the cylinder radius proportional to the magnitude of the inter­action energy. Frameworks were constructed for *E*_ele_ (red cylinders), *E*_dis_ (green) and *E*_tot_ (blue), the scale for tube/cylinder size is 80 and cutoff of 8.00 KJ mol^−1^ was used. Yellow cylinders in Fig. 10 ▸ *a* depicts poor destabilizing positive inter­actions energies in the crystal packing of BF1.

## Synthesis and crystallization   

BF1 and BF2 were synthesised as described in a previous report (Carella *et al.*, 2019 ▸). For both compounds, single crystals suitable for X-ray analysis were obtained by slow evaporation of THF–heptane **(v:v = ?:?)** solutions at room temperature.

## Refinement   

Crystal data, data collection and structure refinement details for BF1 and BF2 are summarized in Table 5 ▸. In both structures, hy­droxy and amine H atoms were found in difference electron-density maps and then freely refined. All the other H atoms were positioned geometrically (C—H = 0.93–0.96 Å) and were refined using a riding model with *U*iso(H) = 1.2*U*_eq_(C) or 1.5*U*_eq_(C-meth­yl). In BF1, the butyl group bound to O5 is disordered over two positions with refined occupancy factors of 0.557 (13) and 0.443 (13). As a result of the brittleness of the crystals, which broke under the cold stream nitro­gen flow, it was not possible to collect data at low temperature. This could explain the rather high *R* values for BF1, where disorder is present.

## Supplementary Material

Crystal structure: contains datablock(s) global, BF1, BF2. DOI: 10.1107/S205698901900728X/dx2017sup1.cif

Structure factors: contains datablock(s) BF1. DOI: 10.1107/S205698901900728X/dx2017BF1sup2.hkl

Supporting information file. DOI: 10.1107/S205698901900728X/dx2017BF1sup4.cml

Structure factors: contains datablock(s) BF2. DOI: 10.1107/S205698901900728X/dx2017BF2sup3.hkl

Supporting information file. DOI: 10.1107/S205698901900728X/dx2017BF2sup5.cml

CCDC references: 1917143, 1917142

Additional supporting information:  crystallographic information; 3D view; checkCIF report

## Figures and Tables

**Figure 3:**
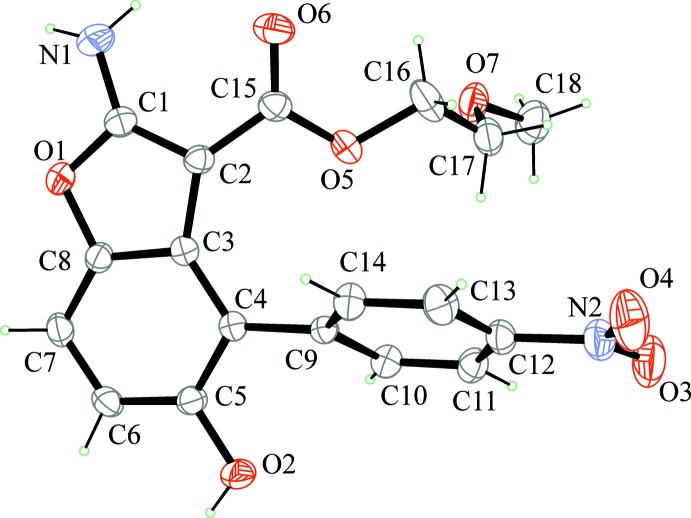
Mol­ecular structure of BF2 with displacement ellipsoids drawn at the 30% probability level.

**Table 1:** Hydrogen-bond geometry (Å, °) for BF1

*D*—H⋯*A*	*D*—H	H⋯*A*	*D*⋯*A*	*D*—H⋯*A*
C7—H7⋯O4^i^	0.93	2.64	3.444 (8)	146
C11—H11⋯O3^ii^	0.93	2.53	3.237 (8)	133
C17*B*—H17*D*⋯O6	0.97	2.74	3.226 (8)	112
N1—H1*A*⋯O6	0.95 (7)	2.15 (7)	2.747 (8)	120 (6)
O2—H2⋯O6^iii^	0.88 (2)	1.94 (3)	2.755 (6)	154 (6)

Symmetry codes: (i) 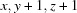; (ii) 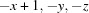; (iii) 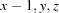.

**Table 2:** Hydrogen-bond geometry (Å, °) for BF2 *Cg*1 and *Cg*2 are the centroids of the O1/C1/C2/C3/C8 and C3–C8 rings, respectively.

*D*—H⋯*A*	*D*—H	H⋯*A*	*D*⋯*A*	*D*—H⋯*A*
C17—H17*A*⋯O3^i^	0.97	2.49	3.366 (3)	150
N1—H1*A*⋯O2^ii^	0.89 (3)	2.14 (3)	3.013 (3)	165 (3)
N1—H1*B*⋯O6	0.83 (3)	2.22 (3)	2.819 (3)	129 (3)
O2—H2*O*⋯O7^iii^	0.88 (3)	1.81 (3)	2.691 (2)	176 (3)
C10—H10⋯*Cg*1^iii^	0.93	2.76	3.521 (3)	139
C11—H11⋯*Cg*2^iii^	0.93	2.80	3.601 (3)	145

Symmetry codes: (i) 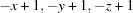; (ii) 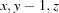; (iii) 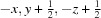.

**Table 3:** Inter­action energies for BF1-molA (kJ mol^−1^) *R* is the distance between mol­ecular centroids (mean atomic position) in Å and *N* is the number of mol­ecules at that distance. Total energies are the sum of the four energy components, scaled according to the appropriate scale factor^(*a*)^.

*N*	symop	*R*	*E*_elec_	*E*_pol_	*E*_energy-dispersive_	*E*_rep_	*E*_total_	inter­action
2	*x*, *y*, *z*	12.04	−9.1	−2.5	−14.1	9.1	−18.1	C7—H7⋯O4^i^
1	−*x*, −*y*, −*z*	10.51	−8.2	−3.0	−11.7	13.1	−12.9	C11—H11⋯O3^ii^
1	−*x*, −*y*, −*z*	11.10	−3.2	−1.9	−12.2	2.8	−13.6	
2	*x*, *y*, *z*	12.43	−3.3	−1.4	−5.4	1.0	−8.6	
1	−*x*, −*y*, −*z*	12.97	8.5	−1.0	−2.3	0.5	6.5	N2—O4⋯O4^iii^
1	−*x*, −*y*, −*z*	5.99	−27.6	−3.7	−67.7	35.8	−68.8	N1—H1*A*⋯O4^iv^
1	−*x*, −*y*, −*z*	6.67	−8.0	−3.0	−43.4	22.8	−34.4	
2	*x*, *y*, *z*	9.44	−42.2	−10.7	−27.7	52.0	−44.5	O2—H2⋯O6^v^
1	−*x*, −*y*, −*z*	5.08	−13.2	−1.5	−76.1	36.5	−58.7	C10—H10⋯O6^vi^
1	−*x*, −*y*, −*z*	9.63	1.5	−1.2	−14.8	3.4	−10.1	
1	−*x*, −*y*, −*z*	11.07	−0.5	−1.9	−14.6	4.5	−11.9	

Notes: (*a*) Energy Model: CE_B3LYP⋯B3LYP/6–31G(d,p) electron densities. Scale factors for benchmarked energy model (Mackenzie *et al.* 2017 ▸): *k*_ele_ = 1.057; *k*_pol_ = 0.740; *k*_energy-dispersive_ = 0.871; *k*_rep_= 0.61. Symmetry codes: (i) *x*, 1 + *y*, 1 + *z*; (ii) 1 − *x*, −*y*, −*z*; (iii) 1 − *x*, −1 − *y*, −*z*; (iv) 2 − *x*, −*y*, 1 − *z*; (v) −1 + *x*, *y*, *z*; (vi) 2 − *x*, 1 − *y*, 1 − *z*.

**Table 4:** Inter­action energies for BF2 (kJ mol^−1^) *R* is the distance between mol­ecular centroids (mean atomic position) in Å and *N* is the number of mol­ecules at that distance. Total energies are the sum of the four energy components, scaled according to the appropriate scale factor^(*a*)^.

*N*	symop	*R*	*E*_elec_	*E*_pol_	*E*_energy-dispersive_	*E*_rep_	*E*_total_	inter­action
1	−*x*, −*y*, −*z*	10.65	−2.1	−0.6	−21.2	12.0	−13.7	
2	−*x*, *y* + 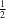 , −*z* + 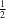	6.41	−70.6	−16.2	−59.8	98.4	−77.9	O2—H2*O*⋯O7^i^
2	*x*, −*y* + 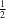 , *z* + 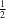	11.49	−4.5	−1.4	−6.2	4.1	−8.6	
2	*x*, *y*, *z*	9.09	−23.5	−7.2	−29.9	35.7	−34.2	N1—H1*A*⋯O2^ii^
1	−*x*, −*y*, −*z*	12.21	−1.4	−0.1	−2.2	0.2	−3.3	
2	*x*, −*y* + 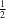 , *z* + 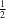	10.11	−1.4	−0.4	−13.6	5.3	−10.4	
2	−*x*, *y* + 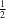 , −*z* + 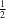	7.42	−8.0	−2.5	−26.4	13.6	−25.0	
1	−*x*, −*y*, −*z*	9.34	−6.5	−0.8	−22.8	12.2	−19.9	
2	*x*, −*y* + 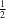 , *z* + 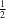	12.67	2.2	−0.3	−3.1	0.6	−0.2	
1	−*x*, −*y*, −*z*	10.57	−17.3	−6.8	−22.2	26.4	−26.3	C17—H17*A*⋯O3^iii^

Notes: (*a*) Energy Model: CE_B3LYP⋯B3LYP/6–31G(d,p) electron densities. Scale factors for benchmarked energy model (Mackenzie *et al.* 2017 ▸): *k*_ele_ = 1.057; *k*_pol_ = 0.740; *k*_energy-dispersive_ = 0.871; *k*_rep_= 0.61. Symmetry codes: (i) −*x*, 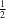 + *y*, 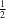 − *z*; (ii) *x*, −1 + *y*, *z*; (iii) 1 − *x*, 1 − *y*, 1 − *z*.

**Table 5:** Experimental details

	BF1	BF2
Crystal data		
Chemical formula	C_19_H_18_N_2_O_6_	C_18_H_16_N_2_O_7_
*M*_r_	370.35	372.33
Crystal system, space group	Triclinic, *P* 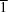	Monoclinic, *P*2_1_/*c*
Temperature (K)	298	298
*a*, *b*, *c* (Å)	9.4420 (16), 9.558 (3), 11.419 (2)	10.263 (2), 9.0860 (8), 20.049 (4)
α, β, γ (°)	110.58 (2), 95.669 (19), 108.863 (19)	90, 111.577 (16), 90
*V* (Å^3^)	886.3 (4)	1738.6 (6)
*Z*	2	4
Radiation type	Mo *K*α	Mo *K*α
μ (mm^−1^)	0.11	0.11
Crystal size (mm)	0.48 × 0.08 × 0.01	0.48 × 0.22 × 0.02

Data collection		
Diffractometer	Bruker-Nonius KappaCCD	Bruker-Nonius KappaCCD
Absorption correction	Multi-scan (*SADABS*; Bruker, 2001 ▸)	Multi-scan (*SADABS*; Bruker, 2001 ▸)
*T*_min_, *T*_max_	0.925, 0.987	0.825, 0.927
No. of measured, independent and observed [*I* > 2σ(*I*)] reflections	5854, 3006, 1212	9595, 3784, 2408
*R*_int_	0.105	0.036
(sin θ/λ)_max_ (Å^−1^)	0.595	0.650

Refinement		
*R*[*F* ^2^ > 2σ(*F* ^2^)], *wR*(*F* ^2^), *S*	0.093, 0.236, 1.21	0.052, 0.139, 1.03
No. of reflections	3006	3784
No. of parameters	274	254
No. of restraints	41	0
H-atom treatment	H atoms treated by a mixture of independent and constrained refinement	H atoms treated by a mixture of independent and constrained refinement
Δρ_max_, Δρ_min_ (e Å^−3^)	0.23, −0.33	0.21, −0.18

Computer programs: *Collect* (Nonius, 1999 ▸), *DIRAX/LSQ* (Duisenberg *et al.*, 2000 ▸), *EVALCCD* (Duisenberg *et al.*, 2003 ▸), *SIR97* (Altomare *et al.*, 1999 ▸), *SHELXL2018* (Sheldrick, 2015 ▸), *ORTEP-3 for Windows* and *WinGX* (Farrugia, 2012 ▸), *Mercury* (Macrae *et al.*, 2006 ▸) and *publCIF* (Westrip, 2010 ▸).

## supplementary crystallographic information

### Butyl 2-amino-5-hydroxy-4-(4-nitrophenyl)benzofuran-3-carboxylate (BF1) Crystal data

**Table:**

C_19_H_18_N_2_O_6_	*Z* = 2
*M**_r_* = 370.35	*F*(000) = 388
Triclinic, *P*1	*D*_x_ = 1.388 Mg m^−^^3^
*a* = 9.4420 (16) Å	Mo *K*α radiation, λ = 0.71073 Å
*b* = 9.558 (3) Å	Cell parameters from 134 reflections
*c* = 11.419 (2) Å	θ = 4.3–18.7°
α = 110.58 (2)°	µ = 0.11 mm^−^^1^
β = 95.669 (19)°	*T* = 298 K
γ = 108.863 (19)°	Needle, yellow
*V* = 886.3 (4) Å^3^	0.48 × 0.08 × 0.01 mm

### Butyl 2-amino-5-hydroxy-4-(4-nitrophenyl)benzofuran-3-carboxylate (BF1) Data collection

**Table:**

Bruker-Nonius KappaCCD diffractometer	3006 independent reflections
Radiation source: normal-focus sealed tube	1212 reflections with *I* > 2σ(*I*)
Graphite monochromator	*R*_int_ = 0.105
Detector resolution: 9 pixels mm^-1^	θ_max_ = 25.0°, θ_min_ = 3.0°
CCD rotation images, thick slices scans	*h* = −11→11
Absorption correction: multi-scan (*SADABS*; Bruker, 2001)	*k* = −11→11
*T*_min_ = 0.925, *T*_max_ = 0.987	*l* = −13→13
5854 measured reflections	

### Butyl 2-amino-5-hydroxy-4-(4-nitrophenyl)benzofuran-3-carboxylate (BF1) Refinement

**Table:**

Refinement on *F*^2^	Primary atom site location: structure-invariant direct methods
Least-squares matrix: full	Secondary atom site location: difference Fourier map
*R*[*F*^2^ > 2σ(*F*^2^)] = 0.093	Hydrogen site location: mixed
*wR*(*F*^2^) = 0.236	H atoms treated by a mixture of independent and constrained refinement
*S* = 1.21	*w* = 1/[σ^2^(*F*_o_^2^) + (0.0652*P*)^2^] where *P* = (*F*_o_^2^ + 2*F*_c_^2^)/3
3006 reflections	(Δ/σ)_max_ < 0.001
274 parameters	Δρ_max_ = 0.23 e Å^−^^3^
41 restraints	Δρ_min_ = −0.33 e Å^−^^3^

### Butyl 2-amino-5-hydroxy-4-(4-nitrophenyl)benzofuran-3-carboxylate (BF1) Special details

**Table:**

Geometry. All esds (except the esd in the dihedral angle between two l.s. planes) are estimated using the full covariance matrix. The cell esds are taken into account individually in the estimation of esds in distances, angles and torsion angles; correlations between esds in cell parameters are only used when they are defined by crystal symmetry. An approximate (isotropic) treatment of cell esds is used for estimating esds involving l.s. planes.
Refinement. The alkyl group at O5 is disordered over two orientations. The two split positions were refined by applying DFIX and SIMU restraints on bond lengths and displacement parameters.

### Butyl 2-amino-5-hydroxy-4-(4-nitrophenyl)benzofuran-3-carboxylate (BF1) Fractional atomic coordinates and isotropic or equivalent isotropic displacement parameters (Å^2^)

**Table:**

	*x*	*y*	*z*	*U*_iso_*/*U*_eq_	Occ. (<1)
C1	1.0745 (7)	0.4807 (8)	0.8031 (6)	0.0708 (17)	
C2	0.9991 (6)	0.3911 (6)	0.6776 (6)	0.0601 (15)	
C3	0.8466 (6)	0.3990 (6)	0.6666 (5)	0.0500 (13)	
C4	0.7108 (6)	0.3317 (5)	0.5697 (5)	0.0478 (13)	
C5	0.5923 (6)	0.3826 (6)	0.6031 (6)	0.0546 (14)	
C6	0.6039 (7)	0.4895 (6)	0.7249 (6)	0.0697 (17)	
H6	0.522787	0.522558	0.741744	0.084*	
C7	0.7321 (7)	0.5481 (6)	0.8216 (6)	0.0671 (17)	
H7	0.740213	0.617845	0.905105	0.081*	
C8	0.8479 (6)	0.4976 (6)	0.7879 (5)	0.0599 (15)	
C9	0.6832 (5)	0.2043 (6)	0.4439 (5)	0.0514 (14)	
C10	0.6654 (7)	0.2268 (7)	0.3306 (6)	0.0727 (18)	
H10	0.671548	0.327540	0.334700	0.087*	
C11	0.6393 (7)	0.1055 (8)	0.2139 (6)	0.0787 (19)	
H11	0.630450	0.122599	0.138677	0.094*	
C12	0.6266 (6)	−0.0422 (7)	0.2106 (6)	0.0657 (16)	
C13	0.6403 (7)	−0.0701 (6)	0.3180 (6)	0.0711 (17)	
H13	0.630802	−0.172387	0.312089	0.085*	
C14	0.6680 (6)	0.0516 (6)	0.4354 (6)	0.0634 (16)	
H14	0.676691	0.032325	0.509609	0.076*	
C15	1.0806 (7)	0.3308 (6)	0.5863 (6)	0.0588 (15)	
C16	1.0804 (7)	0.2212 (8)	0.3631 (6)	0.0825 (19)	
H16A	1.030642	0.226635	0.287168	0.099*	
H16B	1.186378	0.298045	0.390155	0.099*	
C17A	1.0790 (8)	0.0602 (8)	0.3295 (7)	0.103 (2)	0.557 (13)
H17A	0.975065	−0.019365	0.308210	0.123*	0.557 (13)
H17B	1.142348	0.054603	0.398655	0.123*	0.557 (13)
C18A	1.148 (3)	0.036 (2)	0.2114 (16)	0.133 (5)	0.557 (13)
H18A	1.075824	0.031654	0.141926	0.160*	0.557 (13)
H18B	1.241883	0.129730	0.232273	0.160*	0.557 (13)
C19A	1.183 (2)	−0.1118 (16)	0.1655 (16)	0.136 (6)	0.557 (13)
H19A	1.247458	−0.112461	0.235361	0.204*	0.557 (13)
H19B	1.234537	−0.113113	0.097225	0.204*	0.557 (13)
H19C	1.088185	−0.205753	0.134145	0.204*	0.557 (13)
C17B	1.0790 (8)	0.0602 (8)	0.3295 (7)	0.103 (2)	0.443 (13)
H17C	0.972300	−0.014526	0.293379	0.123*	0.443 (13)
H17D	1.112223	0.052835	0.409518	0.123*	0.443 (13)
C18B	1.171 (3)	−0.004 (4)	0.2383 (17)	0.141 (6)	0.443 (13)
H18C	1.270102	0.079386	0.250798	0.169*	0.443 (13)
H18D	1.187648	−0.095011	0.248124	0.169*	0.443 (13)
C19B	1.068 (3)	−0.055 (3)	0.111 (2)	0.162 (7)	0.443 (13)
H19D	0.970732	−0.136990	0.102475	0.244*	0.443 (13)
H19E	1.114365	−0.098113	0.043673	0.244*	0.443 (13)
H19F	1.050918	0.036411	0.105986	0.244*	0.443 (13)
N1	1.2139 (7)	0.5123 (8)	0.8657 (6)	0.094 (2)	
H1A	1.283 (8)	0.475 (8)	0.821 (7)	0.112*	
H1B	1.255 (8)	0.560 (8)	0.944 (7)	0.112*	
N2	0.5962 (7)	−0.1744 (8)	0.0885 (6)	0.0949 (19)	
O1	0.9885 (5)	0.5479 (5)	0.8716 (4)	0.0757 (12)	
O2	0.4613 (5)	0.3189 (5)	0.5099 (4)	0.0776 (13)	
H2	0.406 (6)	0.356 (7)	0.561 (5)	0.093*	
O3	0.5829 (9)	−0.1520 (9)	−0.0083 (6)	0.172 (3)	
O4	0.5908 (9)	−0.3032 (7)	0.0850 (6)	0.164 (3)	
O5	1.0027 (4)	0.2678 (5)	0.4653 (4)	0.0728 (12)	
O6	1.2162 (4)	0.3471 (5)	0.6150 (4)	0.0753 (12)	

### Butyl 2-amino-5-hydroxy-4-(4-nitrophenyl)benzofuran-3-carboxylate (BF1) Atomic displacement parameters (Å^2^)

**Table:**

	*U*^11^	*U*^22^	*U*^33^	*U*^12^	*U*^13^	*U*^23^
C1	0.060 (4)	0.093 (4)	0.064 (5)	0.033 (4)	0.005 (3)	0.035 (3)
C2	0.056 (4)	0.075 (4)	0.055 (4)	0.031 (3)	0.009 (3)	0.029 (3)
C3	0.055 (3)	0.049 (3)	0.054 (4)	0.025 (3)	0.018 (3)	0.023 (3)
C4	0.047 (3)	0.053 (3)	0.047 (3)	0.025 (3)	0.008 (3)	0.018 (3)
C5	0.048 (3)	0.049 (3)	0.063 (4)	0.021 (3)	0.007 (3)	0.018 (3)
C6	0.059 (4)	0.069 (4)	0.082 (5)	0.036 (3)	0.024 (3)	0.019 (3)
C7	0.077 (5)	0.071 (4)	0.059 (4)	0.039 (3)	0.028 (3)	0.020 (3)
C8	0.055 (4)	0.064 (3)	0.053 (4)	0.023 (3)	0.002 (3)	0.018 (3)
C9	0.044 (3)	0.064 (3)	0.054 (4)	0.031 (3)	0.005 (2)	0.024 (3)
C10	0.095 (5)	0.075 (4)	0.064 (5)	0.053 (3)	0.009 (3)	0.030 (3)
C11	0.104 (5)	0.095 (4)	0.042 (4)	0.054 (4)	0.003 (3)	0.025 (3)
C12	0.071 (4)	0.070 (4)	0.046 (4)	0.035 (3)	0.004 (3)	0.007 (3)
C13	0.092 (5)	0.060 (3)	0.065 (5)	0.035 (3)	0.025 (3)	0.023 (3)
C14	0.085 (4)	0.060 (3)	0.063 (4)	0.036 (3)	0.025 (3)	0.036 (3)
C15	0.053 (4)	0.057 (3)	0.066 (4)	0.021 (3)	0.004 (3)	0.027 (3)
C16	0.083 (5)	0.109 (5)	0.082 (5)	0.055 (4)	0.031 (4)	0.049 (4)
C17A	0.110 (6)	0.092 (5)	0.113 (7)	0.059 (4)	0.034 (5)	0.030 (4)
C18A	0.191 (12)	0.135 (11)	0.116 (11)	0.133 (8)	0.046 (9)	0.032 (7)
C19A	0.197 (13)	0.123 (9)	0.116 (11)	0.100 (9)	0.062 (9)	0.039 (8)
C17B	0.110 (6)	0.092 (5)	0.113 (7)	0.059 (4)	0.034 (5)	0.030 (4)
C18B	0.195 (13)	0.137 (12)	0.126 (11)	0.137 (9)	0.038 (10)	0.025 (9)
C19B	0.206 (14)	0.155 (13)	0.127 (13)	0.108 (10)	0.037 (11)	0.023 (10)
N1	0.068 (4)	0.129 (5)	0.071 (4)	0.041 (3)	−0.011 (3)	0.031 (4)
N2	0.118 (5)	0.103 (5)	0.066 (5)	0.064 (4)	0.013 (4)	0.020 (4)
O1	0.080 (3)	0.091 (3)	0.050 (3)	0.040 (2)	0.008 (2)	0.018 (2)
O2	0.063 (3)	0.091 (3)	0.086 (3)	0.049 (2)	0.016 (2)	0.027 (2)
O3	0.267 (8)	0.201 (6)	0.065 (4)	0.165 (6)	−0.002 (5)	0.016 (4)
O4	0.294 (9)	0.092 (3)	0.119 (5)	0.086 (5)	0.094 (5)	0.033 (4)
O5	0.062 (3)	0.102 (3)	0.061 (3)	0.046 (2)	0.019 (2)	0.027 (2)
O6	0.050 (2)	0.100 (3)	0.087 (3)	0.041 (2)	0.013 (2)	0.041 (2)

### Butyl 2-amino-5-hydroxy-4-(4-nitrophenyl)benzofuran-3-carboxylate (BF1) Geometric parameters (Å, º)

**Table:**

C1—N1	1.318 (7)	C16—C17B	1.445 (8)
C1—O1	1.339 (7)	C16—C17A	1.445 (8)
C1—C2	1.355 (8)	C16—O5	1.454 (7)
C2—C15	1.427 (8)	C16—H16A	0.9700
C2—C3	1.462 (7)	C16—H16B	0.9700
C3—C8	1.370 (7)	C17A—C18A	1.530 (9)
C3—C4	1.403 (7)	C17A—H17A	0.9700
C4—C5	1.393 (7)	C17A—H17B	0.9700
C4—C9	1.451 (7)	C18A—C19A	1.484 (9)
C5—O2	1.353 (6)	C18A—H18A	0.9700
C5—C6	1.375 (8)	C18A—H18B	0.9700
C6—C7	1.364 (8)	C19A—H19A	0.9600
C6—H6	0.9300	C19A—H19B	0.9600
C7—C8	1.368 (7)	C19A—H19C	0.9600
C7—H7	0.9300	C17B—C18B	1.516 (10)
C8—O1	1.386 (6)	C17B—H17C	0.9700
C9—C14	1.386 (7)	C17B—H17D	0.9700
C9—C10	1.387 (7)	C18B—C19B	1.487 (10)
C10—C11	1.359 (8)	C18B—H18C	0.9700
C10—H10	0.9300	C18B—H18D	0.9700
C11—C12	1.364 (8)	C19B—H19D	0.9600
C11—H11	0.9300	C19B—H19E	0.9600
C12—C13	1.346 (8)	C19B—H19F	0.9600
C12—N2	1.439 (8)	N1—H1A	0.95 (7)
C13—C14	1.362 (7)	N1—H1B	0.83 (7)
C13—H13	0.9300	N2—O3	1.199 (8)
C14—H14	0.9300	N2—O4	1.202 (7)
C15—O6	1.235 (6)	O2—H2	0.88 (2)
C15—O5	1.319 (6)		
			
N1—C1—O1	116.9 (6)	C17A—C16—H16B	109.0
N1—C1—C2	131.4 (7)	O5—C16—H16B	109.0
O1—C1—C2	111.7 (5)	H16A—C16—H16B	107.8
C1—C2—C15	119.5 (5)	C16—C17A—C18A	101.6 (7)
C1—C2—C3	106.0 (5)	C16—C17A—H17A	111.5
C15—C2—C3	133.8 (5)	C18A—C17A—H17A	111.5
C8—C3—C4	118.1 (5)	C16—C17A—H17B	111.5
C8—C3—C2	105.0 (5)	C18A—C17A—H17B	111.5
C4—C3—C2	136.9 (5)	H17A—C17A—H17B	109.3
C5—C4—C3	116.4 (5)	C19A—C18A—C17A	114.2 (11)
C5—C4—C9	119.3 (4)	C19A—C18A—H18A	108.7
C3—C4—C9	124.0 (5)	C17A—C18A—H18A	108.7
O2—C5—C6	120.6 (5)	C19A—C18A—H18B	108.7
O2—C5—C4	116.7 (5)	C17A—C18A—H18B	108.7
C6—C5—C4	122.7 (5)	H18A—C18A—H18B	107.6
C7—C6—C5	121.2 (5)	C18A—C19A—H19A	109.5
C7—C6—H6	119.4	C18A—C19A—H19B	109.5
C5—C6—H6	119.4	H19A—C19A—H19B	109.5
C6—C7—C8	115.7 (5)	C18A—C19A—H19C	109.5
C6—C7—H7	122.2	H19A—C19A—H19C	109.5
C8—C7—H7	122.2	H19B—C19A—H19C	109.5
C3—C8—C7	125.7 (5)	C16—C17B—C18B	121.7 (13)
C3—C8—O1	110.1 (5)	C16—C17B—H17C	106.9
C7—C8—O1	124.1 (5)	C18B—C17B—H17C	106.9
C14—C9—C10	118.2 (5)	C16—C17B—H17D	106.9
C14—C9—C4	119.0 (5)	C18B—C17B—H17D	106.9
C10—C9—C4	122.8 (5)	H17C—C17B—H17D	106.7
C11—C10—C9	121.7 (5)	C19B—C18B—C17B	101.0 (15)
C11—C10—H10	119.1	C19B—C18B—H18C	111.6
C9—C10—H10	119.1	C17B—C18B—H18C	111.6
C10—C11—C12	117.8 (6)	C19B—C18B—H18D	111.6
C10—C11—H11	121.1	C17B—C18B—H18D	111.6
C12—C11—H11	121.1	H18C—C18B—H18D	109.4
C13—C12—C11	122.4 (5)	C18B—C19B—H19D	109.5
C13—C12—N2	118.2 (6)	C18B—C19B—H19E	109.5
C11—C12—N2	119.4 (6)	H19D—C19B—H19E	109.5
C12—C13—C14	120.0 (5)	C18B—C19B—H19F	109.5
C12—C13—H13	120.0	H19D—C19B—H19F	109.5
C14—C13—H13	120.0	H19E—C19B—H19F	109.5
C13—C14—C9	119.8 (6)	C1—N1—H1A	121 (4)
C13—C14—H14	120.1	C1—N1—H1B	130 (5)
C9—C14—H14	120.1	H1A—N1—H1B	109 (7)
O6—C15—O5	121.8 (5)	O3—N2—O4	120.9 (7)
O6—C15—C2	124.0 (6)	O3—N2—C12	119.1 (7)
O5—C15—C2	114.0 (5)	O4—N2—C12	119.9 (7)
C17B—C16—O5	113.0 (6)	C1—O1—C8	107.1 (5)
C17A—C16—O5	113.0 (6)	C5—O2—H2	96 (4)
C17A—C16—H16A	109.0	C15—O5—C16	119.0 (5)
O5—C16—H16A	109.0		
			
N1—C1—C2—C15	−8.7 (10)	C4—C9—C10—C11	−179.9 (5)
O1—C1—C2—C15	170.4 (5)	C9—C10—C11—C12	1.8 (9)
N1—C1—C2—C3	179.1 (7)	C10—C11—C12—C13	−0.6 (9)
O1—C1—C2—C3	−1.9 (7)	C10—C11—C12—N2	178.9 (6)
C1—C2—C3—C8	1.6 (6)	C11—C12—C13—C14	−0.1 (9)
C15—C2—C3—C8	−169.1 (6)	N2—C12—C13—C14	−179.6 (5)
C1—C2—C3—C4	−176.7 (6)	C12—C13—C14—C9	−0.5 (8)
C15—C2—C3—C4	12.7 (11)	C10—C9—C14—C13	1.7 (8)
C8—C3—C4—C5	5.3 (7)	C4—C9—C14—C13	179.3 (5)
C2—C3—C4—C5	−176.6 (6)	C1—C2—C15—O6	4.2 (9)
C8—C3—C4—C9	−168.8 (5)	C3—C2—C15—O6	173.9 (5)
C2—C3—C4—C9	9.4 (10)	C1—C2—C15—O5	−169.9 (5)
C3—C4—C5—O2	179.7 (5)	C3—C2—C15—O5	−0.2 (9)
C9—C4—C5—O2	−6.0 (7)	O5—C16—C17A—C18A	173.0 (11)
C3—C4—C5—C6	−1.7 (8)	C16—C17A—C18A—C19A	171.5 (17)
C9—C4—C5—C6	172.7 (5)	O5—C16—C17B—C18B	−171.7 (11)
O2—C5—C6—C7	176.5 (5)	C16—C17B—C18B—C19B	−81 (2)
C4—C5—C6—C7	−2.1 (9)	C13—C12—N2—O3	179.7 (7)
C5—C6—C7—C8	2.0 (9)	C11—C12—N2—O3	0.1 (10)
C4—C3—C8—C7	−5.8 (9)	C13—C12—N2—O4	−3.4 (10)
C2—C3—C8—C7	175.6 (6)	C11—C12—N2—O4	177.1 (7)
C4—C3—C8—O1	177.8 (4)	N1—C1—O1—C8	−179.4 (6)
C2—C3—C8—O1	−0.9 (6)	C2—C1—O1—C8	1.3 (7)
C6—C7—C8—C3	2.0 (9)	C3—C8—O1—C1	−0.2 (6)
C6—C7—C8—O1	177.9 (5)	C7—C8—O1—C1	−176.7 (6)
C5—C4—C9—C14	−106.8 (6)	O6—C15—O5—C16	−2.1 (8)
C3—C4—C9—C14	67.1 (7)	C2—C15—O5—C16	172.2 (5)
C5—C4—C9—C10	70.6 (7)	C17B—C16—O5—C15	85.4 (7)
C3—C4—C9—C10	−115.4 (6)	C17A—C16—O5—C15	85.4 (7)
C14—C9—C10—C11	−2.4 (8)		

### Butyl 2-amino-5-hydroxy-4-(4-nitrophenyl)benzofuran-3-carboxylate (BF1) Hydrogen-bond geometry (Å, º)

**Table:**

*D*—H···*A*	*D*—H	H···*A*	*D*···*A*	*D*—H···*A*
C7—H7···O4^i^	0.93	2.64	3.444 (8)	146
C11—H11···O3^ii^	0.93	2.53	3.237 (8)	133
C17*B*—H17*D*···O6	0.97	2.74	3.226 (8)	112
N1—H1*A*···O6	0.95 (7)	2.15 (7)	2.747 (8)	120 (6)
O2—H2···O6^iii^	0.88 (2)	1.94 (3)	2.755 (6)	154 (6)

Symmetry codes: (i) *x*, *y*+1, *z*+1; (ii) −*x*+1, −*y*, −*z*; (iii) *x*−1, *y*, *z*.

### 2-Methoxyethyl 2-amino-5-hydroxy-4-(4-nitrophenyl)benzofuran-3-carboxylate (BF2) Crystal data

**Table:**

C_18_H_16_N_2_O_7_	*F*(000) = 776
*M**_r_* = 372.33	*D*_x_ = 1.422 Mg m^−^^3^
Monoclinic, *P*2_1_/*c*	Mo *K*α radiation, λ = 0.71073 Å
*a* = 10.263 (2) Å	Cell parameters from 72 reflections
*b* = 9.0860 (8) Å	θ = 3.0–20.3°
*c* = 20.049 (4) Å	µ = 0.11 mm^−^^1^
β = 111.577 (16)°	*T* = 298 K
*V* = 1738.6 (6) Å^3^	Plate, yellow
*Z* = 4	0.48 × 0.22 × 0.02 mm

### 2-Methoxyethyl 2-amino-5-hydroxy-4-(4-nitrophenyl)benzofuran-3-carboxylate (BF2) Data collection

**Table:**

Bruker-Nonius KappaCCD diffractometer	3784 independent reflections
Radiation source: normal-focus sealed tube	2408 reflections with *I* > 2σ(*I*)
Graphite monochromator	*R*_int_ = 0.036
Detector resolution: 9 pixels mm^-1^	θ_max_ = 27.5°, θ_min_ = 3.0°
CCD rotation images, thick slices scans	*h* = −8→13
Absorption correction: multi-scan (*SADABS*; Bruker, 2001)	*k* = −11→10
*T*_min_ = 0.825, *T*_max_ = 0.927	*l* = −26→23
9595 measured reflections	

### 2-Methoxyethyl 2-amino-5-hydroxy-4-(4-nitrophenyl)benzofuran-3-carboxylate (BF2) Refinement

**Table:**

Refinement on *F*^2^	0 restraints
Least-squares matrix: full	Hydrogen site location: mixed
*R*[*F*^2^ > 2σ(*F*^2^)] = 0.052	H atoms treated by a mixture of independent and constrained refinement
*wR*(*F*^2^) = 0.139	*w* = 1/[σ^2^(*F*_o_^2^) + (0.058*P*)^2^ + 0.521*P*] where *P* = (*F*_o_^2^ + 2*F*_c_^2^)/3
*S* = 1.03	(Δ/σ)_max_ < 0.001
3784 reflections	Δρ_max_ = 0.21 e Å^−^^3^
254 parameters	Δρ_min_ = −0.18 e Å^−^^3^

### 2-Methoxyethyl 2-amino-5-hydroxy-4-(4-nitrophenyl)benzofuran-3-carboxylate (BF2) Special details

**Table:**

Geometry. All esds (except the esd in the dihedral angle between two l.s. planes) are estimated using the full covariance matrix. The cell esds are taken into account individually in the estimation of esds in distances, angles and torsion angles; correlations between esds in cell parameters are only used when they are defined by crystal symmetry. An approximate (isotropic) treatment of cell esds is used for estimating esds involving l.s. planes.

### 2-Methoxyethyl 2-amino-5-hydroxy-4-(4-nitrophenyl)benzofuran-3-carboxylate (BF2) Fractional atomic coordinates and isotropic or equivalent isotropic displacement parameters (Å^2^)

**Table:**

	*x*	*y*	*z*	*U*_iso_*/*U*_eq_	
C1	0.1522 (2)	−0.1808 (2)	0.16883 (11)	0.0445 (5)	
C2	0.1956 (2)	−0.0554 (2)	0.20982 (10)	0.0373 (5)	
C3	0.1095 (2)	0.0632 (2)	0.16748 (10)	0.0341 (4)	
C4	0.1002 (2)	0.2168 (2)	0.17400 (10)	0.0344 (4)	
C5	−0.0070 (2)	0.2886 (2)	0.11922 (11)	0.0406 (5)	
C6	−0.0987 (2)	0.2149 (2)	0.05940 (11)	0.0461 (5)	
H6	−0.169033	0.267025	0.024435	0.055*	
C7	−0.0860 (2)	0.0655 (2)	0.05158 (11)	0.0450 (5)	
H7	−0.145055	0.015044	0.011512	0.054*	
C8	0.0173 (2)	−0.0043 (2)	0.10548 (10)	0.0390 (5)	
C9	0.2033 (2)	0.3040 (2)	0.23255 (10)	0.0340 (4)	
C10	0.1601 (2)	0.3939 (2)	0.27675 (11)	0.0420 (5)	
H10	0.065367	0.399616	0.269523	0.050*	
C11	0.2554 (2)	0.4745 (2)	0.33092 (11)	0.0467 (5)	
H11	0.225981	0.534073	0.360395	0.056*	
C12	0.3945 (2)	0.4653 (2)	0.34070 (11)	0.0444 (5)	
C13	0.4412 (2)	0.3798 (2)	0.29739 (12)	0.0503 (6)	
H13	0.535893	0.376423	0.304353	0.060*	
C14	0.3444 (2)	0.2992 (2)	0.24337 (11)	0.0440 (5)	
H14	0.374444	0.240694	0.213767	0.053*	
C15	0.2933 (2)	−0.0684 (2)	0.28310 (11)	0.0424 (5)	
C16	0.3911 (3)	0.0471 (3)	0.39702 (12)	0.0675 (7)	
H15A	0.476930	0.089810	0.395981	0.081*	
H15B	0.411377	−0.052314	0.415599	0.081*	
C17	0.3393 (3)	0.1360 (3)	0.44439 (12)	0.0639 (7)	
H17A	0.418292	0.173723	0.484457	0.077*	
H17B	0.285814	0.219097	0.417820	0.077*	
C18	0.2178 (3)	0.1191 (3)	0.52447 (15)	0.0807 (9)	
H18A	0.301484	0.142483	0.564594	0.121*	
H18B	0.160476	0.054063	0.539727	0.121*	
H18C	0.166857	0.207917	0.505670	0.121*	
N1	0.1990 (3)	−0.3192 (2)	0.17972 (13)	0.0662 (7)	
H1A	0.137 (3)	−0.388 (3)	0.1558 (15)	0.079*	
H1B	0.258 (3)	−0.334 (3)	0.2202 (16)	0.079*	
N2	0.4964 (3)	0.5494 (2)	0.39927 (11)	0.0620 (6)	
O1	0.04482 (16)	−0.15477 (14)	0.10650 (8)	0.0487 (4)	
O2	−0.01457 (18)	0.43883 (16)	0.12485 (9)	0.0579 (5)	
H2O	−0.094 (3)	0.471 (3)	0.0929 (14)	0.069*	
O3	0.4543 (2)	0.6155 (2)	0.44027 (10)	0.0856 (6)	
O4	0.6174 (2)	0.5507 (3)	0.40383 (12)	0.0956 (7)	
O5	0.28607 (16)	0.04351 (16)	0.32517 (7)	0.0519 (4)	
O6	0.37098 (18)	−0.17310 (18)	0.30529 (9)	0.0630 (5)	
O7	0.25432 (19)	0.04923 (17)	0.47021 (8)	0.0628 (5)	

### 2-Methoxyethyl 2-amino-5-hydroxy-4-(4-nitrophenyl)benzofuran-3-carboxylate (BF2) Atomic displacement parameters (Å^2^)

**Table:**

	*U*^11^	*U*^22^	*U*^33^	*U*^12^	*U*^13^	*U*^23^
C1	0.0499 (13)	0.0369 (11)	0.0459 (12)	−0.0015 (9)	0.0168 (11)	0.0021 (9)
C2	0.0385 (12)	0.0333 (10)	0.0406 (11)	−0.0025 (8)	0.0150 (9)	0.0018 (8)
C3	0.0328 (11)	0.0374 (11)	0.0341 (10)	−0.0029 (8)	0.0144 (8)	0.0005 (8)
C4	0.0327 (11)	0.0378 (11)	0.0334 (10)	−0.0007 (8)	0.0130 (8)	−0.0013 (8)
C5	0.0406 (12)	0.0346 (11)	0.0448 (12)	0.0016 (8)	0.0135 (10)	0.0010 (9)
C6	0.0425 (13)	0.0489 (13)	0.0385 (11)	0.0023 (9)	0.0051 (10)	0.0056 (9)
C7	0.0481 (13)	0.0488 (13)	0.0334 (11)	−0.0085 (9)	0.0095 (9)	−0.0038 (9)
C8	0.0459 (12)	0.0353 (11)	0.0369 (11)	−0.0046 (8)	0.0165 (9)	−0.0030 (8)
C9	0.0346 (11)	0.0314 (10)	0.0344 (10)	−0.0002 (7)	0.0109 (8)	0.0015 (8)
C10	0.0382 (12)	0.0436 (12)	0.0459 (12)	−0.0009 (9)	0.0177 (10)	−0.0042 (9)
C11	0.0574 (15)	0.0434 (12)	0.0426 (12)	−0.0045 (10)	0.0221 (11)	−0.0073 (9)
C12	0.0489 (14)	0.0439 (12)	0.0357 (11)	−0.0119 (9)	0.0099 (10)	−0.0002 (9)
C13	0.0348 (12)	0.0612 (14)	0.0528 (14)	−0.0076 (10)	0.0137 (11)	−0.0026 (11)
C14	0.0367 (12)	0.0501 (12)	0.0463 (12)	−0.0006 (9)	0.0165 (10)	−0.0078 (9)
C15	0.0418 (12)	0.0391 (12)	0.0447 (12)	−0.0043 (9)	0.0142 (10)	0.0069 (9)
C16	0.0584 (17)	0.089 (2)	0.0386 (13)	−0.0056 (13)	−0.0011 (11)	0.0059 (12)
C17	0.0819 (19)	0.0544 (15)	0.0396 (13)	−0.0198 (12)	0.0039 (12)	−0.0003 (11)
C18	0.097 (2)	0.082 (2)	0.0644 (18)	−0.0044 (16)	0.0318 (17)	−0.0191 (15)
N1	0.0802 (17)	0.0332 (11)	0.0703 (15)	−0.0010 (10)	0.0100 (12)	0.0010 (10)
N2	0.0671 (16)	0.0586 (13)	0.0475 (12)	−0.0200 (11)	0.0061 (11)	−0.0018 (10)
O1	0.0610 (10)	0.0359 (8)	0.0435 (8)	−0.0051 (7)	0.0126 (7)	−0.0066 (6)
O2	0.0537 (10)	0.0383 (9)	0.0629 (11)	0.0095 (7)	−0.0005 (8)	0.0010 (7)
O3	0.1101 (17)	0.0828 (14)	0.0548 (12)	−0.0276 (12)	0.0195 (12)	−0.0262 (10)
O4	0.0596 (14)	0.1190 (18)	0.0861 (15)	−0.0335 (12)	0.0009 (11)	−0.0229 (12)
O5	0.0540 (10)	0.0558 (10)	0.0352 (8)	0.0042 (7)	0.0040 (7)	0.0019 (7)
O6	0.0615 (11)	0.0517 (10)	0.0639 (11)	0.0120 (8)	0.0091 (9)	0.0131 (8)
O7	0.0778 (12)	0.0530 (10)	0.0515 (10)	−0.0191 (8)	0.0165 (9)	−0.0113 (8)

### 2-Methoxyethyl 2-amino-5-hydroxy-4-(4-nitrophenyl)benzofuran-3-carboxylate (BF2) Geometric parameters (Å, º)

**Table:**

C1—N1	1.335 (3)	C12—N2	1.468 (3)
C1—O1	1.349 (3)	C13—C14	1.380 (3)
C1—C2	1.380 (3)	C13—H13	0.9300
C2—C15	1.447 (3)	C14—H14	0.9300
C2—C3	1.453 (3)	C15—O6	1.216 (2)
C3—C8	1.397 (3)	C15—O5	1.341 (2)
C3—C4	1.409 (3)	C16—O5	1.448 (3)
C4—C5	1.397 (3)	C16—C17	1.486 (4)
C4—C9	1.487 (3)	C16—H15A	0.9700
C5—O2	1.375 (2)	C16—H15B	0.9700
C5—C6	1.393 (3)	C17—O7	1.408 (3)
C6—C7	1.378 (3)	C17—H17A	0.9700
C6—H6	0.9300	C17—H17B	0.9700
C7—C8	1.361 (3)	C18—O7	1.424 (3)
C7—H7	0.9300	C18—H18A	0.9600
C8—O1	1.394 (2)	C18—H18B	0.9600
C9—C14	1.384 (3)	C18—H18C	0.9600
C9—C10	1.392 (3)	N1—H1A	0.89 (3)
C10—C11	1.375 (3)	N1—H1B	0.83 (3)
C10—H10	0.9300	N2—O4	1.212 (3)
C11—C12	1.370 (3)	N2—O3	1.218 (3)
C11—H11	0.9300	O2—H2O	0.88 (3)
C12—C13	1.376 (3)		
			
N1—C1—O1	116.06 (19)	C12—C13—H13	120.8
N1—C1—C2	131.5 (2)	C14—C13—H13	120.8
O1—C1—C2	112.45 (17)	C13—C14—C9	121.09 (19)
C1—C2—C15	119.26 (18)	C13—C14—H14	119.5
C1—C2—C3	105.70 (17)	C9—C14—H14	119.5
C15—C2—C3	134.37 (17)	O6—C15—O5	122.9 (2)
C8—C3—C4	118.08 (17)	O6—C15—C2	123.5 (2)
C8—C3—C2	105.17 (16)	O5—C15—C2	113.51 (17)
C4—C3—C2	136.74 (17)	O5—C16—C17	109.8 (2)
C5—C4—C3	116.75 (17)	O5—C16—H15A	109.7
C5—C4—C9	119.87 (17)	C17—C16—H15A	109.7
C3—C4—C9	123.23 (17)	O5—C16—H15B	109.7
O2—C5—C6	120.48 (18)	C17—C16—H15B	109.7
O2—C5—C4	116.78 (18)	H15A—C16—H15B	108.2
C6—C5—C4	122.66 (18)	O7—C17—C16	110.3 (2)
C7—C6—C5	120.60 (19)	O7—C17—H17A	109.6
C7—C6—H6	119.7	C16—C17—H17A	109.6
C5—C6—H6	119.7	O7—C17—H17B	109.6
C8—C7—C6	116.59 (19)	C16—C17—H17B	109.6
C8—C7—H7	121.7	H17A—C17—H17B	108.1
C6—C7—H7	121.7	O7—C18—H18A	109.5
C7—C8—O1	124.28 (18)	O7—C18—H18B	109.5
C7—C8—C3	125.21 (18)	H18A—C18—H18B	109.5
O1—C8—C3	110.50 (17)	O7—C18—H18C	109.5
C14—C9—C10	118.59 (18)	H18A—C18—H18C	109.5
C14—C9—C4	120.45 (17)	H18B—C18—H18C	109.5
C10—C9—C4	120.95 (17)	C1—N1—H1A	115.4 (19)
C11—C10—C9	120.97 (19)	C1—N1—H1B	114 (2)
C11—C10—H10	119.5	H1A—N1—H1B	122 (3)
C9—C10—H10	119.5	O4—N2—O3	123.5 (2)
C12—C11—C10	118.8 (2)	O4—N2—C12	118.3 (2)
C12—C11—H11	120.6	O3—N2—C12	118.1 (2)
C10—C11—H11	120.6	C1—O1—C8	106.12 (15)
C11—C12—C13	122.05 (19)	C5—O2—H2O	109.6 (17)
C11—C12—N2	118.8 (2)	C15—O5—C16	116.75 (18)
C13—C12—N2	119.2 (2)	C17—O7—C18	113.83 (19)
C12—C13—C14	118.5 (2)		
			
N1—C1—C2—C15	−11.7 (4)	C3—C4—C9—C10	−124.6 (2)
O1—C1—C2—C15	169.66 (17)	C14—C9—C10—C11	−1.2 (3)
N1—C1—C2—C3	176.4 (2)	C4—C9—C10—C11	179.96 (18)
O1—C1—C2—C3	−2.2 (2)	C9—C10—C11—C12	0.2 (3)
C1—C2—C3—C8	2.4 (2)	C10—C11—C12—C13	1.0 (3)
C15—C2—C3—C8	−167.7 (2)	C10—C11—C12—N2	−179.02 (19)
C1—C2—C3—C4	−176.8 (2)	C11—C12—C13—C14	−1.2 (3)
C15—C2—C3—C4	13.2 (4)	N2—C12—C13—C14	178.81 (19)
C8—C3—C4—C5	3.6 (3)	C12—C13—C14—C9	0.2 (3)
C2—C3—C4—C5	−177.3 (2)	C10—C9—C14—C13	0.9 (3)
C8—C3—C4—C9	−171.97 (18)	C4—C9—C14—C13	179.83 (19)
C2—C3—C4—C9	7.1 (3)	C1—C2—C15—O6	22.3 (3)
C3—C4—C5—O2	−178.79 (17)	C3—C2—C15—O6	−168.7 (2)
C9—C4—C5—O2	−3.0 (3)	C1—C2—C15—O5	−155.14 (19)
C3—C4—C5—C6	−2.0 (3)	C3—C2—C15—O5	13.9 (3)
C9—C4—C5—C6	173.74 (19)	O5—C16—C17—O7	83.6 (2)
O2—C5—C6—C7	176.1 (2)	C11—C12—N2—O4	−173.8 (2)
C4—C5—C6—C7	−0.5 (3)	C13—C12—N2—O4	6.2 (3)
C5—C6—C7—C8	1.4 (3)	C11—C12—N2—O3	5.4 (3)
C6—C7—C8—O1	179.86 (19)	C13—C12—N2—O3	−174.5 (2)
C6—C7—C8—C3	0.5 (3)	N1—C1—O1—C8	−177.8 (2)
C4—C3—C8—C7	−3.0 (3)	C2—C1—O1—C8	1.1 (2)
C2—C3—C8—C7	177.62 (19)	C7—C8—O1—C1	−178.9 (2)
C4—C3—C8—O1	177.49 (16)	C3—C8—O1—C1	0.6 (2)
C2—C3—C8—O1	−1.8 (2)	O6—C15—O5—C16	9.3 (3)
C5—C4—C9—C14	−118.9 (2)	C2—C15—O5—C16	−173.19 (18)
C3—C4—C9—C14	56.5 (3)	C17—C16—O5—C15	−157.75 (19)
C5—C4—C9—C10	59.9 (3)	C16—C17—O7—C18	170.9 (2)

### 2-Methoxyethyl 2-amino-5-hydroxy-4-(4-nitrophenyl)benzofuran-3-carboxylate (BF2) Hydrogen-bond geometry (Å, º)

*Cg*1 and *Cg*2 are the centroids of the O1/C1/C2/C3/C8 and C3–C8 rings, respectively.

**Table:**

*D*—H···*A*	*D*—H	H···*A*	*D*···*A*	*D*—H···*A*
C17—H17*A*···O3^i^	0.97	2.49	3.366 (3)	150
N1—H1*A*···O2^ii^	0.89 (3)	2.14 (3)	3.013 (3)	165 (3)
N1—H1*B*···O6	0.83 (3)	2.22 (3)	2.819 (3)	129 (3)
O2—H2*O*···O7^iii^	0.88 (3)	1.81 (3)	2.691 (2)	176 (3)
C10—H10···*Cg*1^iii^	0.93	2.76	3.521 (3)	139
C11—H11···*Cg*2^iii^	0.93	2.80	3.601 (3)	145

Symmetry codes: (i) −*x*+1, −*y*+1, −*z*+1; (ii) *x*, *y*−1, *z*; (iii) −*x*, *y*+1/2, −*z*+1/2.
